# Tumour antigenicity in ovarian cancer.

**DOI:** 10.1038/bjc.1975.144

**Published:** 1975-08

**Authors:** L. Levin, J. E. McHardy, O. M. Curling, C. N. Hudson

## Abstract

The blastogenic response to a crude cell extract of ovarian cancer cells has been studied in 48 patients with ovarian cancer (9, autologous, 39 allogeneic), in 26 female controls matched for age and in 18 female patients with other types of cancer in remission from disease. The responses in ovarian cancer patients in remission and relapse were considered separately. The blastogenic responses to cell extracts of foetal ovary, foetal lung, foetal liver and normal adult ovary were also assessed in a proportion of all 3 groups. The blastogenic responses to ovarian cancer and foetal ovary cell extracts were found to be significantly greater in the ovarian cancer patients in remission than in the controls, but the responses to ovarian cancer extract were not greater in the relapse group or in patients with other cancers. As a blastogenic response to normal ovarian extract was also present in some of these patients, the data so far do not support the hypothesis of a tumour specific antigen. This tumour associated response may be occurring to determinants in foetal or adult ovarian tissue to which the patient becomes sensitized in malignant disease. The response is complex and the nature of the antigen requires further analysis.


*Br. J. Cancer* (1975) **32**, 152

### TUMOUR ANTIGENICITY IN OVARIAN CANCER

L. LEVIN, J. E. McHARDY, O. M. CURLING AND C. N. HUDSON

*From the Williamson Laboratory, Medical College of St Bartholomew's Hospital, London*

Received 7 March 1975. Accepted 16 April 1975

**Summary.—The blastogenic response to a crude cell extract of ovarian cancer cells has been studied in 48 patients with ovarian cancer (9, autologous, 39 allogeneic), in 26 female controls matched for age and in 18 female patients with other types of cancer in remission from disease. The responses in ovarian cancer patients in remission and relapse were considered separately. The blastogenic responses to cell extracts of foetal ovary, foetal lung, foetal liver and normal adult ovary were also assessed in a proportion of all 3 groups.**

**The blastogenic responses to ovarian cancer and foetal ovary cell extracts were found to be significantly greater in the ovarian cancer patients in remission than in the controls, but the responses to ovarian cancer extract were not greater in the relapse group or in patients with other cancers. As a blastogenic response to normal ovarian extract was also present in some of these patients, the data so far do not support the hypothesis of a tumour specific antigen. This tumour associated response may be occurring to determinants in foetal or adult ovarian tissue to which the patient becomes sensitized in malignant disease. The response is complex and the nature of the antigen requires further analysis.**

AN IMMUNE response which appears to be directed towards tumour antigens has been demonstrated *in vitro* for several human tissues (Hellström *et al.*, 1971). Attention in recent years has been centred on the cell mediated immune response to tumours and this response appears to be instrumental in facilitating tumour rejection (Burnet, 1968).

Cultured peripheral lymphocytes will transform into lymphoblasts when challenged *in vitro* by an antigen to which the host has previously been sensitized (Coulson and Chalmers, 1967; Bouveng, Gardwell and Low, 1967). We have determined whether the lymphocytes from patients with ovarian cancer will transform when challenged with ovarian cancer cell extracts and whether there is any correlation between the extent of disease and the ability of the lymphocytes to transform *in vitro*.

Much attention has been given to embryonic antigens in recent years and the hypothesis that "tumour specific antigens" are re-expressed embryonic antigens has been derived from studies on viral and chemically induced animal tumours (Coggin, Ambrose and Anderson, 1970; Coggin *et al.*, 1971; Baldwin, Glaves and Vose, 1972). We have therefore also looked at the blastogenic response to a cell extract of foetal ovary to see whether an oncofoetal antigen might be at least partly responsible for this blastogenic response.

To assess whether the blastogenic responses were in any way caused by normal ovarian constituents in the tumour or foetal extracts, lymphocytes from patients in all 3 groups were challenged with cell extracts from normal ovarian tissue.

### MATERIALS AND METHODS

Tumour and normal ovarian cell extracts (CE) were obtained by a modified method

Abbreviations: FCS, Foetal Calf Serum; AS, Autologous Serum; CE, Cell Extract, ct/min.; S.I. Stimulation Index (S.I. = Stimulated ct/min/Unstimulated ct/min).

described by Oren and Herberman (1971). Approximately 5 g of fresh tumour was disaggregated in 0·14 mol/l sodium chloride (1 ml/g wet tissue) by high speed homogenization, frozen rapidly in liquid nitrogen and then thawed; finally hypotonic saline was added. The tumour suspension was centrifuged at 400 *g* (4 °C) after each of these procedures, the supernatant fluid from each stage being pooled and centrifuged at 105,000 *g* at 4 °C for one hour. The protein content of the resultant supernatant was estimated by the technique of Lowry, Rosebrough and Farr (1952).

Preparation of the foetal tissue extracts was modified slightly. The initial stage was as described above but in the final stage the extract was centrifuged at 3000 *g* at 4 °C for one hour and the resulting supernatant used to assess blastogenic responses; this modification was introduced to procure a larger yield of protein than would have resulted from ultracentrifugation.

Lymphocytes were prepared by layering defibrinated blood over Ficoll/Triosil (Bøyum, 1968) and subsequently resuspending in Tissue Culture Medium 199 containing 12·5% of autologous or foetal calf serum, at a concentration of $10^6$ lymphocytes/ml. Then 100 μg of the ovarian cancer, normal ovary or foetal ovary cell extract (CE) was added to appropriate tubes containing $1 \times 10^6$ lymphocytes, the amount of CE for optimal blastogenesis being determined by dose-response assay. Each experiment was performed in duplicate.

The lymphocyte cultures were incubated for 5 days at 37 °C in 5% $CO_2$, pulsed with 1·0 μCi $^{125}$I UDR (5 iodo-2 deoxyuridine) for 4 h and harvested by washing twice with Hanks' medium and once with 10% cold trichloracetic acid. The $^{125}$I UDR incorporation into the acid insoluble fraction was then measured by using a Wilj gamma counter.

Lymphocyte responses in 48 patients with ovarian cancer, 18 females with other cancers in remission from disease and 26 controls were challenged with CE from serous papillary cystadenocarcinoma of the ovary. The controls were normal non-pregnant females aged between 32 and 74 years, mostly awaiting elective surgery for prolapse.

Eighteen of the patients with ovarian cancer, 13 with other cancers and 13 controls were tested for responses to foetal ovary; in these patients blastogenic responses to foetal liver or lung (from the same foetus) were also assessed to evaluate the possibility of transplantation allo-antigenic responses.

Responses to normal ovarian extracts were assessed in 11 of the ovarian cancer patients, 7 normal controls and 8 patients with other cancers, to determine whether any responses to ovarian cancer or ovarian foetal extracts could be attributed to sensitization to normal tissue determinants.

No patients had had any chemotherapy or radiotherapy for at least 2 months before these tests were performed, and none of the allogeneic tests were conducted on patients who had received a blood transfusion for at least 6 months before the test was carried out.

### RESULTS

### *Dose response assays for ovarian cancer cell extract (CE)*

$1 \times 10^6$ lymphocytes were stimulated with doses of CE varying between 50 and 200 μg wherever this was possible (Fig. 1). From the data obtained we calculated that the optimal dose for blastogenic stimulation was generally 100 μg.

### *Blastogenic responses*

The blastogenic responses to 100 μg CE for patients with ovarian cancer both in remission and relapse and for patients with other cancers are shown in Fig. 2, the response being expressed for each patient as the ct/min difference between stimulated and unstimulated lymphocytes. These data are further subdivided into ct/min differences for lymphocytes incubated in FCS or AS. A stimulatory effect was recorded above the zero line, an inhibitory effect below it. Responses to normal ovarian cell extract are also shown on these graphs. The means of difference between stimulated and unstimulated responses are shown at the bottom of Fig. 2.

The blastogenic responses to 100 μg CE for patients with ovarian cancer in remission are shown in Table I, these responses being expressed as the stimul-

FIG. 1.—Blastogenic responses to varying doses of ovarian cancer cell extracts.

ation index. Responses to autologous extracts and responses from patients with endometrioid cancer of the ovary are also shown in this Table.

The $\chi^2$ and $P$ values for responses in the 3 groups of patients compared with normal controls are shown in Table II. Significant differences were found between the response of ovarian cancer remission patients and the normal controls in both AS and FCS ($P < 0 \cdot 01$); there was no significant difference between the response of ovarian cancer relapse patients and the normal controls.

The percentage error between any two measurements of the same response was approximately 12%.

Figure 3 records the ct/min differences for the blastogenic responses to foetal ovarian and other foetal tissue (lung or liver). The means of the difference between stimulated and unstimulated responses are shown at the bottom of this graph.

A response to foetal ovary extract was present in the ovarian cancer group which

FIG. 2.—Comparison of blastogenic responses to ovarian cancer CE in ovarian cancer remission and relapse patients, normal controls and female patients with other cancers.

was not present in the control group. Although there was no great difference in response to the extract between the ovarian cancer group and patients with other cancers, responses in ovarian cancer groups were higher and occurred in more patients.

There was no significant difference in response to ovarian cancer extract compared with the response to normal adult ovary or foetal ovary in any groups.

DISCUSSION

Evidence for both " tumour associated " and " tumour specific " antigens in ovarian cancer has been claimed by other workers (Levi, Keller and Mandl, 1969; Hellström *et al.*, 1971; Di Saia *et al.*, 1971; Bhattacharya and Barlow, 1973; Knaupf and Urbach, 1974). However, from our data it would appear that the antigenic determinants present in ovarian cancer are really rather complex.

TABLE I.—*Blastogenic Response of Ovarian Cancer Patients in Remission to Ovarian Tumour Cell Extract*

| AS | | FCS | | Stimulation Index (stimulated/unstimulated) | |
|---|---|---|---|---|---|
| Unstimulated (ct/min) | Stimulated 100 μg CE (ct/min) | Unstimulated (ct/min) | Stimulated 100μg CE (ct/min) | AS | FCS |
| *10350 | 23280 | 9870 | 9420 | 2·2 | 1·0 |
| *3521 | 3735 | 3315 | 3651 | 1·1 | 1·1 |
| *1890 | 2400 | 2050 | 4440 | 1·3 | 2·2 |
| *5940 | 9025 | — | — | 1·5 | |
| *2340 | 2670 | 1380 | 1890 | 1·1 | 1·4 |
| *4095 | 5790 | 1560 | 3960 | 1·5 | 2·5 |
| *3120 | 3210 | 2850 | 3840 | 1·0 | 1·3 |
| †7020 | 7500 | 6060 | 8940 | 1·1 | 1·5 |
| †3000 | 4000 | 2900 | 8000 | 1·4 | 3·1 |
| †7200 | 13080 | 9240 | 17100 | 1·9 | 1·8 |
| †2350 | 2601 | 2520 | 4260 | 1·1 | 1·7 |
| 4480 | 4200 | 3020 | 2540 | 0·9 | 0·8 |
| 2430 | 3840 | 3570 | 2040 | 1·6 | 0·6 |
| 4320 | 2870 | 3000 | 4301 | 0·7 | 1·4 |
| 3240 | 4950 | 2200 | 2640 | 1·5 | 1·2 |
| 3510 | 2700 | 2250 | 2640 | 0·8 | 1·2 |
| 3300 | 4680 | 2280 | 4440 | 1·4 | 2·0 |
| 2010 | 1800 | 1740 | 2310 | 0·9 | 1·3 |
| 2480 | 2840 | 3100 | 2700 | 1·2 | 0·9 |
| 6940 | 7400 | 6060 | 8940 | 1·0 | 1·5 |
| 1320 | 2700 | 1530 | 1380 | 2·0 | 0·9 |
| 2100 | 3120 | 1740 | 3180 | 1·5 | 1·8 |
| 2340 | 2070 | 990 | 2640 | 0·9 | 2·7 |
| 2760 | 3300 | — | — | 2·2 | |
| | | 3630 | 4260 | | 1·2 |
| 2640 | 2070 | 4460 | 4980 | 0·8 | 1·1 |

* Autologous CE.

† Endometrioid carcinoma of the ovary stimulated by serous papillary cystadenocarcinoma CE.

TABLE II.—*To show $\chi^2$ and Probability Levels, Comparing Ovarian Cancer Patients and Other Cancer Patients with Normal Controls in AS and FCS*

| | OV Ca remission | | OV Ca relapse | | Other cancers | |
|---|---|---|---|---|---|---|
| | AS | FCS | AS | FCS | AS | FCS |
| Normal | $\chi^2 = 9{\cdot}818$ | $P = 11{\cdot}289$ | $\chi^2 = 8{\cdot}70$ | $\chi^2 = 4{\cdot}791$ | $\chi^2 = 5{\cdot}176$ | $\chi^2 = 1{\cdot}976$ |
| Controls | $P = 0{\cdot}007$ | $P = 0{\cdot}003$ | $P = 0{\cdot}6$ | $P = 0{\cdot}09$ | $P$ 0·08 | $P = 0{\cdot}3$ |

The blastogenic response to ovarian cancer extract shows that ovarian cancer patients in remission are sensitized to a determinant (s) in the extract when normal controls are not. While some of the patients with progressive disease are also sensitized to the determinants, the responses in this group are not significantly different from the controls. Incubating lymphocytes in FCS only marginally improved responses in the relapse group of patients.

The impaired blastogenic response to CE in relapse patients may reflect a general impairment in the ability of lymphocytes from these patients to transform to any appropriate antigenic stimulus. Impaired cell mediated immunity has been demonstrated *in vivo* by delayed skin hypersensitivity tests in patients with ovarian cancer who are in relapse (Khoo and Mackay, 1974). *In vitro* lymphocyte impairment has been demonstrated in non-lymphoid tumours by Catalona, Sample and Chretien (1973). However, Di Saia *et al.* (1971) demonstrated *in vitro* lymphocyte cytoxicity on ovarian cancer cells from relapse patients

FIG. 3.—Comparison of blastogenic responses to foetal ovary and foetal lung or liver CE in ovarian cancer patients, normal controls and female patients with other cancers.

when the lymphocytes were incubated in FCS.

In experiments where allogeneic CE was used, histocompatibility (HLA) antigen could have induced transformation, but the fact that there was no significant transformation to the CE in the control group, and the fact that transformation to autologous CE occurred in 6 of 11 patients, suggests that HLA does not contribute to the blastogenic response in this system. This is consistent with the findings of Mavligit *et al.* (1973) and Gutterman *et al.* (1972) who have found that solubilized antigens stimulate *in vitro* blastogenesis only if the lymphocytes have been sensitized to the antigen *in vivo*.

The tendency for lymphocytes from patients with endometrioid ovarian cancer to transform when challenged with CE of serous papillary carcinoma requires further consideration. If the response is due to a tumour specific antigen this would suggest that the antigen is possibly organ-specific and this could include sensitization to normal ovarian determinants.

The ovarian tumours dealt with in this paper share a common embryological origin and the possibility that an oncofoetal antigen was responsible for the organ specific responses therefore prompted us to investigate this aspect further. This idea has been substantiated to some extent by the blastogenic response to ovarian foetal ovary extract and has not hitherto been reported. From our data, the response to foetal ovary appears to be tumour associated, although the responses are greater in the ovarian cancer patients.

In view of the fact that we have also demonstrated blastogenic responses to normal ovarian cell extract, the existence of ovarian cancer oncofoetal antigens should be regarded with some reserve until determinants in normal adult ovary responsible for blastogenesis can be excluded from a purified extract of foetal ovary.

A cell mediated response to normal breast tissue has been demonstrated in patients with breast cancer (Alford, Hollinshead and Herberman, 1973) and to normal lung tissue in patients with lung cancer (Hollinshead, Stewart and Herberman, 1974). It appears that the same holds true for ovarian cancer patients in their response to normal ovary extracts. This would fit in with the postulate that exposure to normal cell constituents by a break down of the basement membrane in tumours can sensitize the cancer patients to these normal cell products (Hall, 1974). Antigens which could be responsible for the blastogenic effect of normal ovarian extract include normal tissue antigen described by Dickinson *et al.* (1974) and oncofoetal antigens which may occur in normal tissue (Burtin, 1974). More sophisticated methods are required to detect an autoimmune element in the host response to tumour and we are investigating this possibility further.

*Conclusion*

Patients with ovarian cancer in remission have a blastogenic response to autologous and allogeneic ovarian cancer cell extract which is significantly greater than in control patients. The response is reduced in relapse patients. This implies sensitization to a determinant (s) in the extract which is not demonstrable in relapse; incubating the lymphocytes in FCS only marginally improves the response. Although the major response has been in patients with ovarian cancer, it does not appear to be specific to these tumours. Our work suggests that there are determinants which are shared by ovarian tumours of different histology but of the same postulated embryological origin; since we have demonstrated a blastogenic response to foetal ovary extract, these shared antigens may be oncofoetal. Nevertheless, because we have also demonstrated blastogenic responses to normal ovary, it will be necessary to eliminate the determinants responsible for this before the exact nature of any "tumour associated" antigen can be established.

This work is financed by the Cancer Research Campaign (GYNI) by whom L. L., J. E. McH. and O. M. C. are supported. The original pilot studies were carried out by Dr R. Powles at the Chester Beatty Institute and we are indebted to him and to Dr G. Currie for continuing advice and assistance. We are grateful also to Professor N. A. Mitchison for advice on the establishment of the blastogenic assay. We are particularly grateful for colleagues from the Association of Obstetricians and Gynaecologists of the N. E. Thames Region and others who have allowed us access to patients and who have provided fresh tumour material for the investigation. The Regional Ovarian Cancer Study is supported by the Regional Health Authority through funds for Locally Organized Clinical Research, after an initial grant from the Peel Medical Trust.

### REFERENCES

ALFORD, C., HOLLINSHEAD, A. C. & HERBERMAN, R. B. (1973) Delayed Cutaneous Hypersensitivity Reactions to Extracts of Malignant and Normal Human Breast Cells. *Ann. Surg.*, **178**, 20.

BALDWIN, R. W., GLAVES, D. & VOSE, B. M. (1972) Embryonic Antigen Expression in Chemically Induced Rat Hepatomas and Sarcomas. *Int. J. Cancer*, **10**, 23.

BHATTACHARYA, M. & BARLOW, J. J. (1973) Immunologic Studies of Human Serous Cystadenocarcinoma of Ovary. *Cancer N.Y.*, **31**, 1588.

BOUVENG, R., GARDWELL, S. & LOW, B. (1967) The Effect of Antigens on DNA-synthesis in Cultured Lymphocytes. *Scand. J. Haematol.*, **4**, 125.

BØYUM, A. (1968) Separation of Leucocytes from Blood and Bone Marrow. *Scand. J. clin. Lab. Invest.*, **21**, Suppl., 97.

BURNET, F. M. (1968) Evolution of the Immune Process in Vertebrates. *Nature, Lond.*, **218**, 426.

BURTIN, P. (1974) Carcino-embryonic Antigens. In *Progress in Immunology* II Vol. 3. Eds. L. Brent and J. Holborow. Amsterdam: North Holland Publishing Co.

CATALONA, W. J., SAMPLE, W. F. & CHRETIEN, P. B. (1972) Lymphocyte Reactivity in Cancer Patients: Correlation with Tumor Histology and Clinical Stage. *Cancer N.Y.*, **31**, 65.

COGGIN, J. H., AMBROSE, K. R. & ANDERSON, N. G. (1970) Fetal Antigen Capable of Inducing Transplantation Immunity against SV40 Hamster Tumor Cells. *J. Immun.*, **105**, 524.

COGGIN, J. H., AMBROSE, K. R., BELLONEY, B. B. & ANDERSON, N. G. (1971) Tumour Immunity in Hamsters Immunized with Fetal Tissue. *J. Immun.*, **107**, 526.

COULSON, A. S. & CHALMERS, D. G. (1967) Response of Human Blood Lymphocytes to Tuberculin and P.P.D. in Tissue Culture. *Immunology*, **12**, 416.

DICKINSON, J. P., MCDERMOTT, J. R., SMITH, J. K. & CASPARY, E. A. (1974) A Common Tumour Specific Antigen. II Further Characterization of the Whole Antigen and of a Cross-reacting Antigen of Normal Tissues. *Br. J. Cancer*, **29**, 425.

DI SAIA, P. J., RUTLEDGE, F. N., SMITH, J. P. & SINKOVICS, J. G. (1971) Cell mediated Immune Reaction to Two Gynecologic Malignant Tumors. *Cancer N.Y.*, **28**, 1129.

GUTTERMAN, J. V., MAVLIGIT, G., MCCREDIE, K. B., BODEY, G. P., FREIREICH, E. J. & HERSH, E. M. (1972) Antigen Solubilized from Human Leukemia—Lymphocytes Stimulation. *Science N.Y.*, **177** 1114.

HALL, T. C. (1974) Oncocognitive Immunity. *Cancer chemother. Rep.*, **58**, 441.

HELLSTRÖM, I., HELLSTRÖM, K. E., SJÖGREN, H. O. & WARNER, G. A. (1971) Demonstration of Cell Mediated Immunity to Human Neoplasm of Various Histological Types. *Int. J. Cancer*, **7**, 1.

HOLLINSHEAD, A. C, STEWART, T. H. M. & HERBERMAN, R. B. (1974) Delayed Hypersensitivity Reactions to Soluble Membrane Antigens of Human Malignant Lung Cells. *J. natn. Cancer Inst.*, **52**, 327.

KHOO, S. K. & MACKAY, E. V. (1974) Relation of Cell Mediated Immunity in Women with Genital Tract Cancer to Origin, Histology, Clinical Stage and Subsequent Behaviour of Neoplasm. *J. Obstet. Gynaec. Br. Cwlth*, **81**, 229.

KNAUPF, S & URBACH, G. I. (1974) Ovarian Tumor-specific Antigens. *Am. J. Obstet. Gynec.*, **119**, 966.

LEVI, M. M., KELLER, S. & MANDL, I. (1969) Antigenicity of a Papillary Serous Cystadenocarcinoma Tissue Homogenate and its Fractions. *Am. J. Obstet. Gynec.*, **105**, 856.

LOWRY, O. H., ROSEBROUGH, N. J. & FARR, A. L. (1952) Protein Measurement with Folin Phenol Reagent. *J. biol. Chem.*, **193**, 265.

MAVLIGIT, G., AMBUS, U., GUTTERMAN, J. U., MCBRIDE, C. M. & HERSH, E. M. (1973) Antigens solubilized from Human Solid Tumours—Lymphocyte Stimulation and Cutaneous Delayed Hypersensitivity. *Nature, New Biol.*, **243**, 188.

OREN, M. E. & HERBERMAN, R. B. (1971) Delayed Cutaneous Hypersensitivity Reactions to Membrane Extracts of Human Tumor Cells. *Clin. & exp. Immunol.*, **9**, 45.


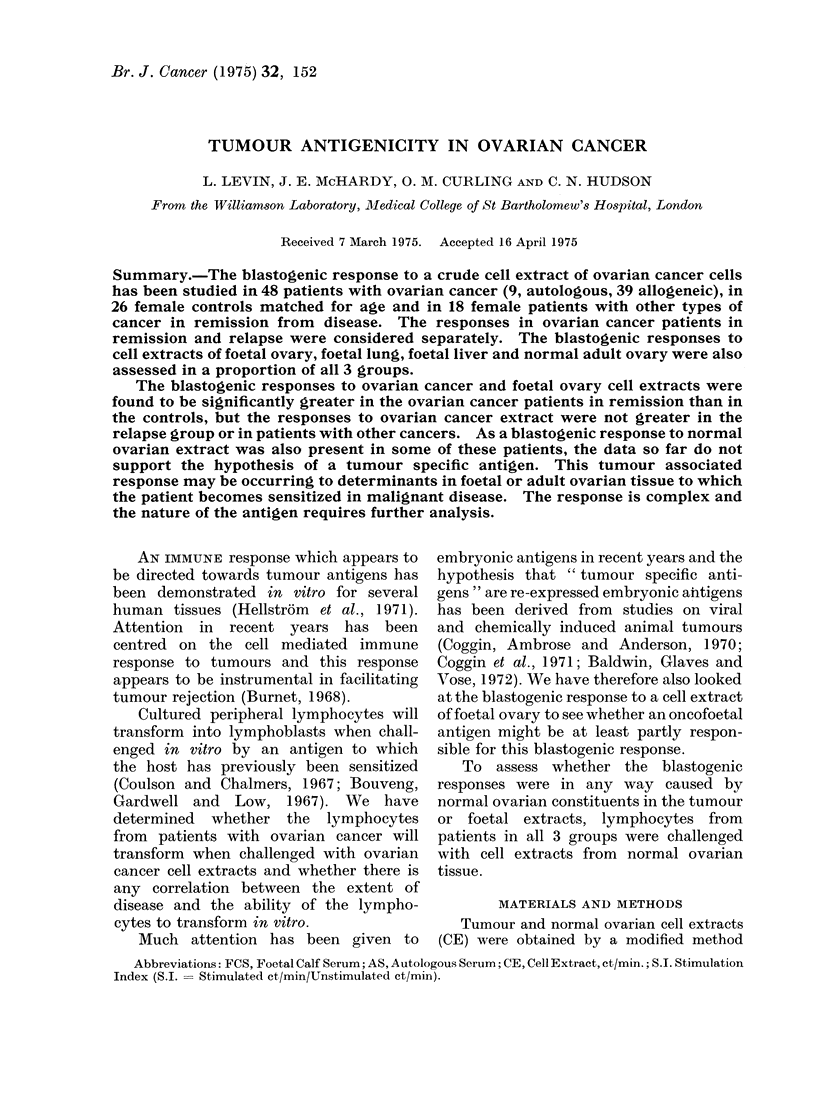

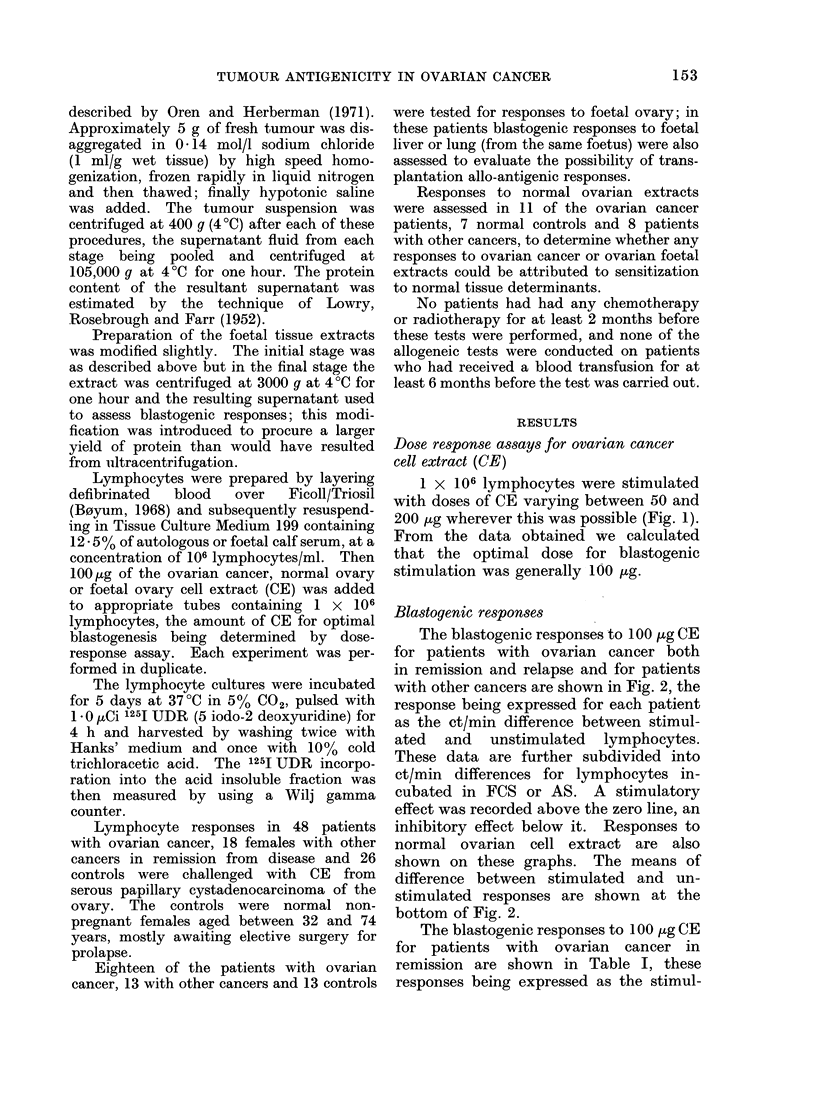

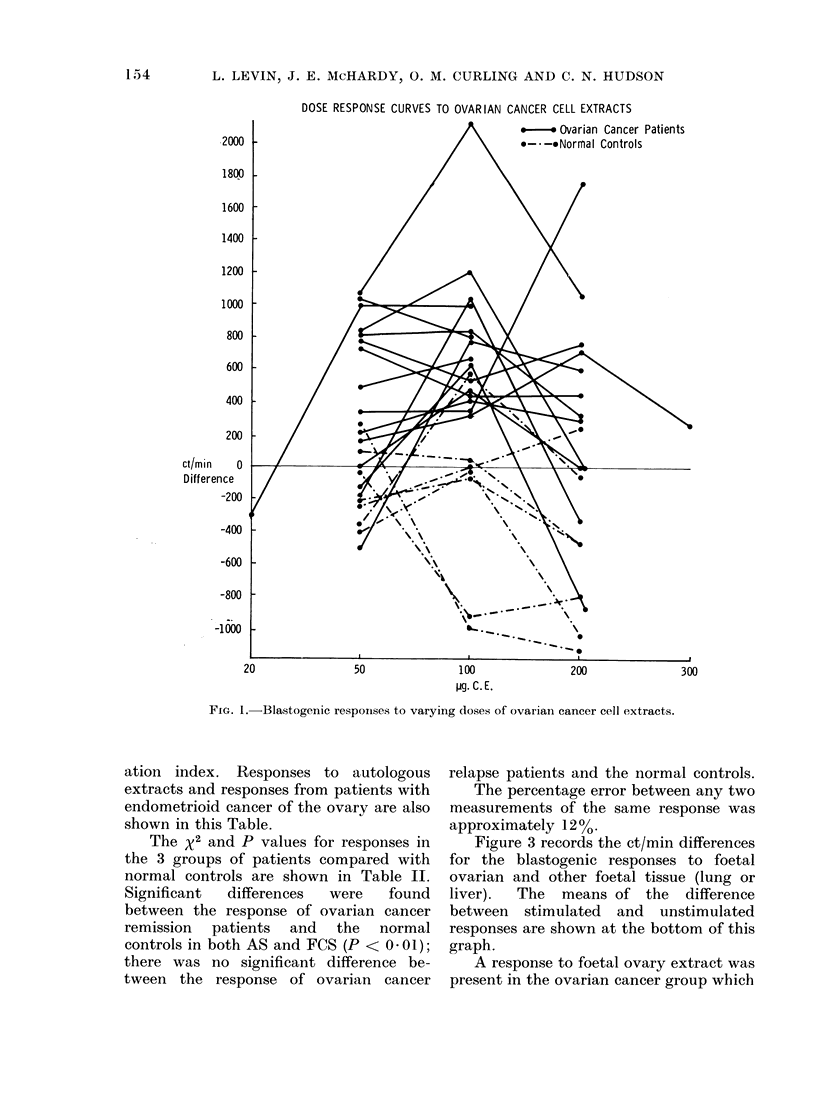

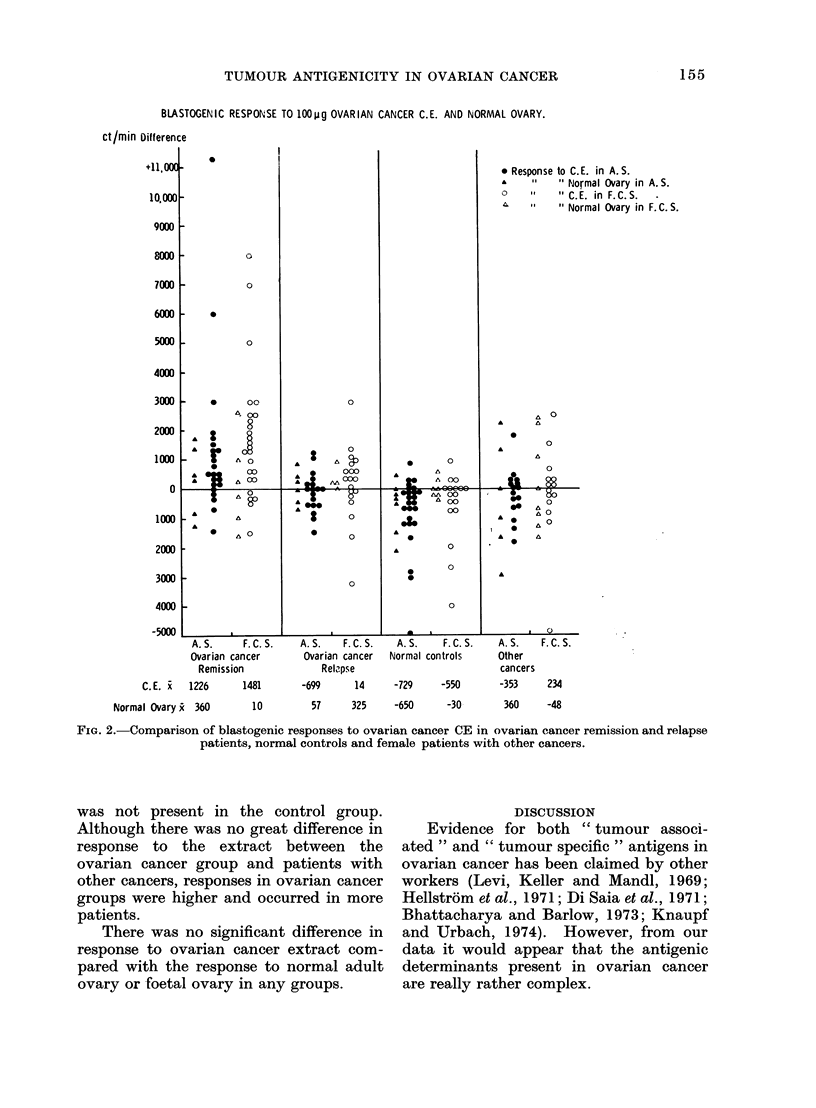

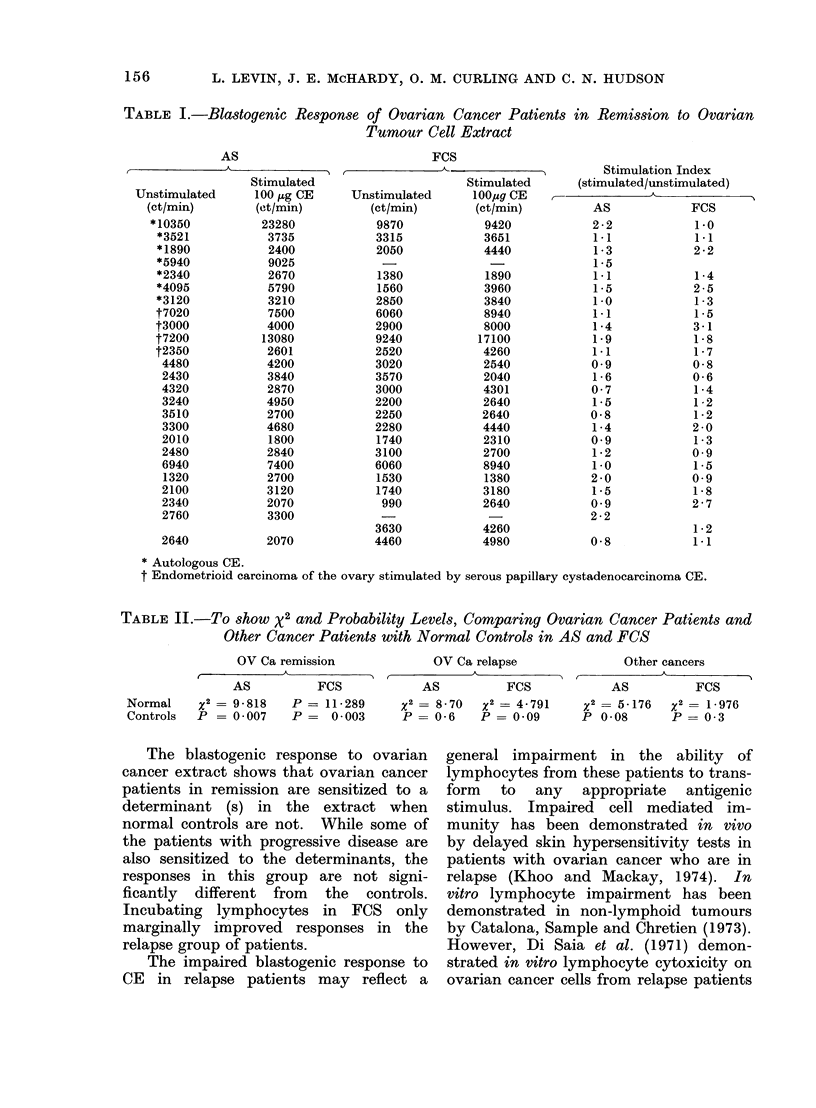

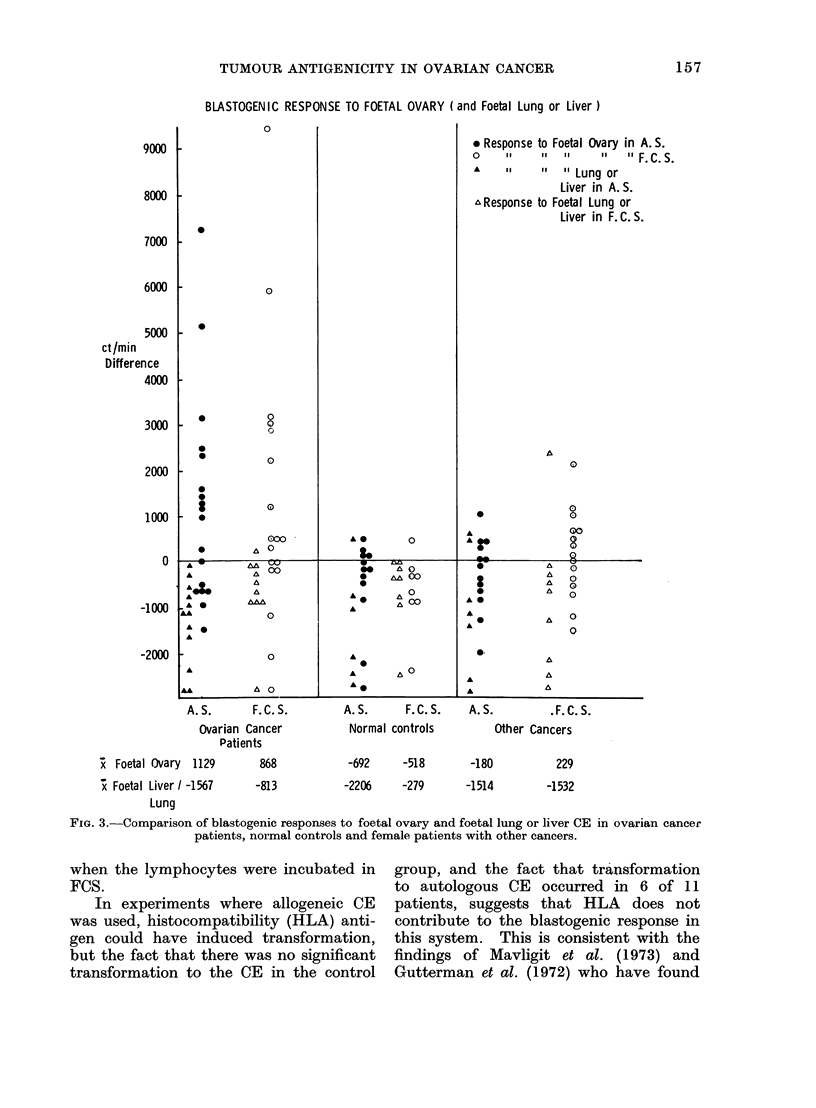

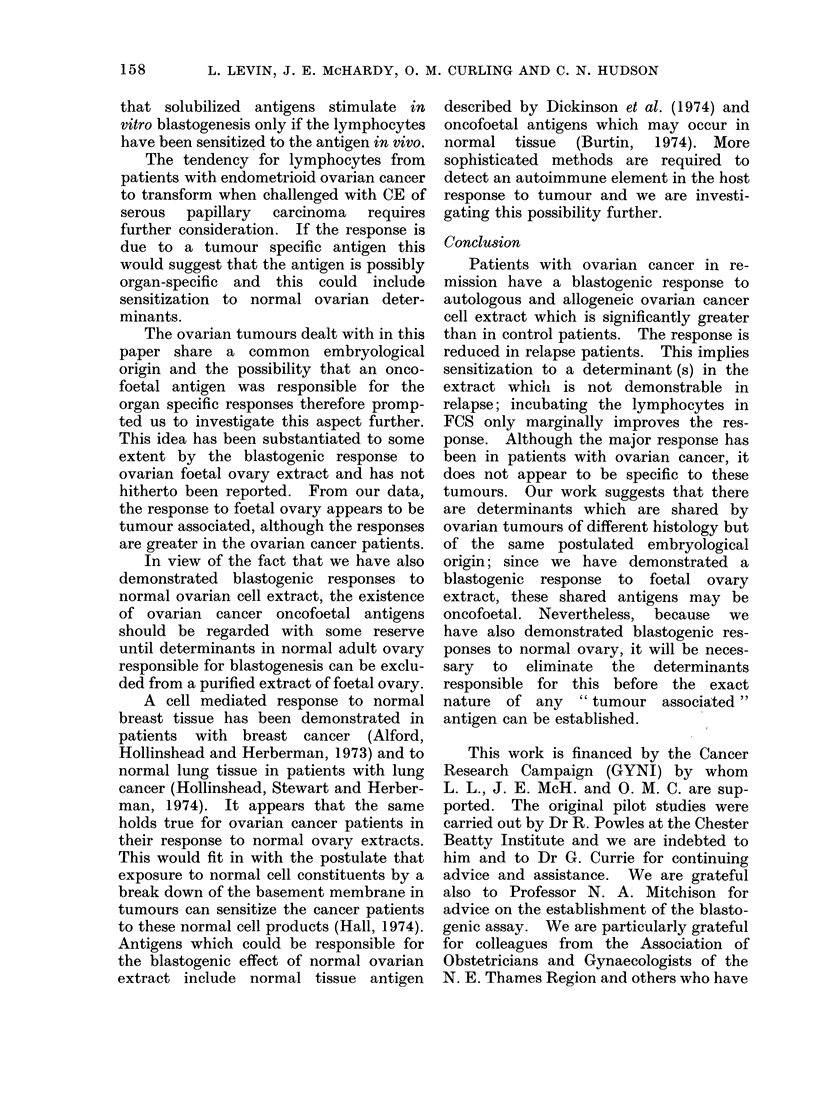

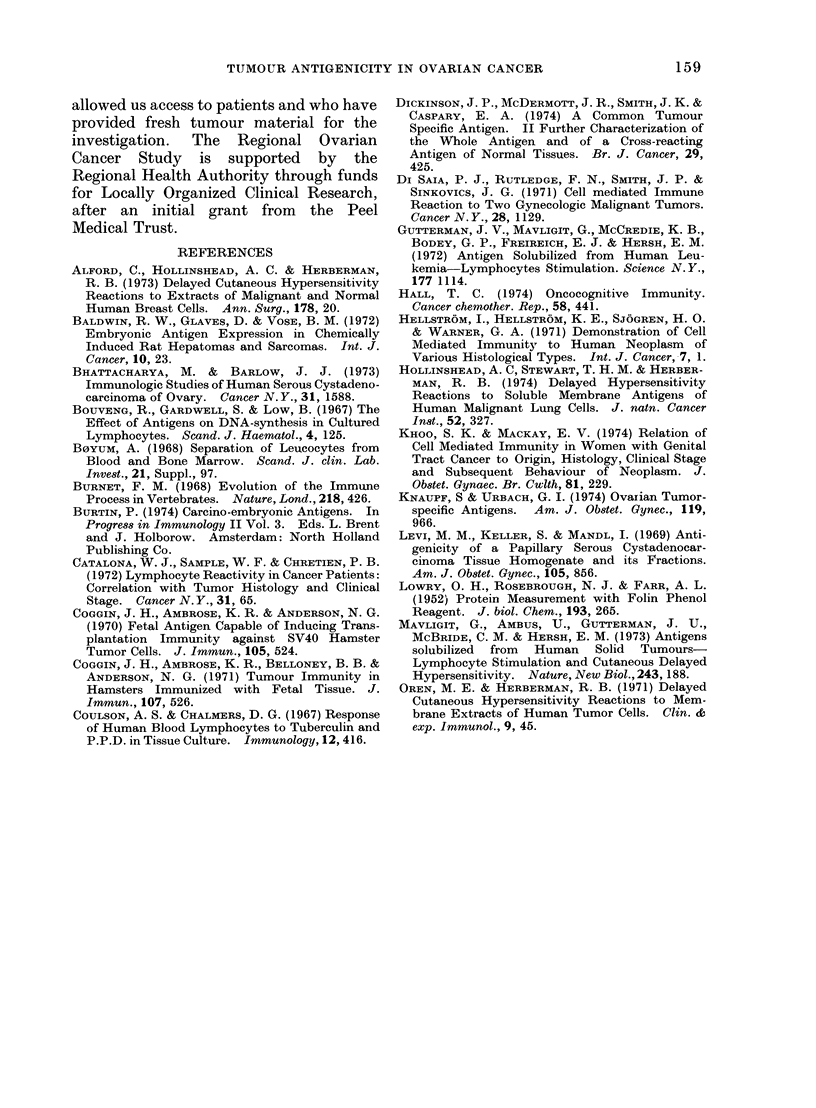


## References

[OCR_00903] Alford C., Hollinshead A. C., Heberman R. B. (1973). Delayed cutaneous hypersensitivity reactions to extracts of malignant and normal breast cells.. Ann Surg.

[OCR_00940] Catalona W. J., Sample W. F., Chretien P. B. (1973). Lymphocyte reactivity in cancer patients: correlation with tumor histology and clinical stage.. Cancer.

[OCR_00946] Coggin J. H., Ambrose K. R., Anderson N. G. (1970). Fetal antigen capable of inducing transplantation immunity against SV40 hamster tumor cells.. J Immunol.

[OCR_00952] Coggin J. H., Ambrose K. R., Bellomy B. B., Anderson N. G. (1971). Tumor immunity in hamsters immunized with fetal tissues.. J Immunol.

[OCR_00971] DiSala P. J., Rutledge F. N., Smith J. P., Sinkovics J. G. (1971). Cell-mediated immune reaction to two gynecologic malignant tumors.. Cancer.

[OCR_00963] Dickinson J. P., McDermott J. R., Smith J. K., Caspary E. A. (1974). A common tumour specific antigen. II. Further characterization of the whole antigen and of a cross-reacting antigen of normal tissues.. Br J Cancer.

[OCR_01000] Khoo S. K., Mackay E. V. (1974). Relation of cell-mediated immunity in women with genital tract cancer to origin, histology, clinical stage and subsequent behaviour of neoplasm.. J Obstet Gynaecol Br Commonw.

[OCR_01007] Knauf S., Urbach G. I. (1974). Ovarian tumor-specific antigens.. Am J Obstet Gynecol.

[OCR_01018] LOWRY O. H., ROSEBROUGH N. J., FARR A. L., RANDALL R. J. (1951). Protein measurement with the Folin phenol reagent.. J Biol Chem.

[OCR_01012] Levi M. M., Keller S., Mandl I. (1969). Antigenicity of a papillary serous cystadenocarcinoma tissue homogenate and its fractions.. Am J Obstet Gynecol.

[OCR_01023] Mavligit G. M., Ambus U., Gutterman J. U., Hersh E. M., McBride C. M. (1973). Antigen solubilized from human solid tumours: lymphocyte stimulation and cutaneous delayed hypersensitivity.. Nat New Biol.

[OCR_01030] Oren M. E., Herberman R. B. (1971). Delayed cutaneous hypersensitivity reactions to membrane extracts of human tumour cells.. Clin Exp Immunol.

